# Short‐term GAA loading: Responders versus nonresponders analysis

**DOI:** 10.1002/fsn3.1744

**Published:** 2020-06-26

**Authors:** Sergej M. Ostojic

**Affiliations:** ^1^ Faculty of Sport and Physical Education University of Novi Sad Novi Sad Serbia; ^2^ Faculty of Health Sciences University of Pecs Pecs Hungary

**Keywords:** creatine, guanidinoacetic acid, MR spectroscopy, muscle, non‐responders, supplementation

## Abstract

Dietary guanidinoacetic acid (GAA) has been suggested to be advantageous for favorable changes in tissue bioenergetics in terms of responder versus nonresponder performance, yet no studies so far explored the proportion of two distinct populations following short‐term GAA intervention. A secondary analysis of previously completed guanidinoacetic acid (GAA) trials has been carried out in aim to classify individuals into responders and nonresponders using cut‐off criteria for an increase in intramuscular creatine. A total of 30 individuals (mean age = 34.5 years, women 66.7%) who were supplemented with up to 3 g/day of GAA for at least 28 days with total muscle creatine evaluated using 1.5 T magnetic resonance spectroscopy studies were included in this examination. Pre–post measures included total creatine content (creatine plus phosphocreatine) determined from the quadriceps muscle, with participants were classified by arbitrary cut‐off points in three categories, including responders (>10% increase in total creatine content at follow‐up), quasi‐responders (5%–10% increase), and nonresponders (<5% increase in total intramuscular creatine at postadministration). An average change in total creatine content after GAA supplementation was 22.9%, with 13.3% participants were categorized as nonresponders, 6.6% as quasi‐responders, and 80.0% as responders (*p* < .001). A fairly high prevalence of individuals sensitive to dietary GAA advances this innovative agent as a rather effective tool to improve muscle creatine levels for at least 10% or more during 28‐day loading.

## Graphical abstract

A secondary analysis of previously completed guanidinoacetic acid (GAA) trials has been carried out in aim to classify individuals into responders and nonresponders using cut‐off criteria for an increase in intramuscular creatine. An average change in total creatine content after GAA supplementation was 22.9%, with 13.3% participants were categorized as nonresponders, 6.6% as quasi‐responders, and 80.0% as responders (*p* < .001), thus advancing this innovative agent as an effective tool to improve muscle creatine.

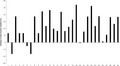

## 1 INTRODUCTION

Guanidinoacetic acid (GAA) is an endogenous amino acid derivative that acts as a direct precursor of creatine, an organic compound serving as a rapidly mobilizable reserve of high‐energy phosphates for energy‐demanding tissues such as the brain or muscle. Supplemental GAA has been introduced to human nutrition and medicine ~70 years ago as an effective intervention to facilitate creatine‐mediated energy provision in different health conditions (Borsook & Borsook, 1951). Recent human studies have reaffirmed favorable characteristics of this investigational compound after short‐ and medium‐term administration, including improved work capacity (Ostojic, Stojanovic, et al., 2016), advantageous tissue bioenergetics (Ostojic, Ostojic, Drid, & Vranes, 2016), or acceptable safety (Ostojic, Niess, Stojanovic, & Obrenovic, 2013). Furthermore, dietary GAA has been anecdotally suggested to be advantageous in terms of responder versus nonresponder performance, yet no studies so far explored the proportion of two distinct populations following GAA intervention. In this report, a secondary analysis of previously completed short‐term GAA trials has been carried out in aim to classify individuals into responders and nonresponders using cut‐off criteria for an increase in intramuscular creatine, and to describe their fundamental metabolic profiles.

## 2 METHODS

A total of 30 individuals (mean age = 34.5 years, 20 women) who were supplemented with up to 3 g/day of GAA for at least 28 days with total muscle creatine evaluated using 1.5 T magnetic resonance spectroscopy (MRS) studies were included in this examination. Pre–post measures included total creatine content (creatine plus phosphocreatine) determined from the quadriceps muscle MRS spectra using TARQUIN 4.3.10 software. All participants were classified by arbitrary cut‐off points in three categories, including responders (>10% increase in total creatine content [mM] at follow‐up), quasi‐responders (5%–10% increase), and nonresponders (<5% increase in total intramuscular creatine at postadministration). Detailed information of the methods used, including participants details and ethical issues, has been described previously (Ostojic, Drid, & Ostojic, 2016; Ostojic, Ostojic, et al., 2016; Ostojic, Stojanovic, et al., 2016).

## 3 RESULTS

**FIGURE 1:**
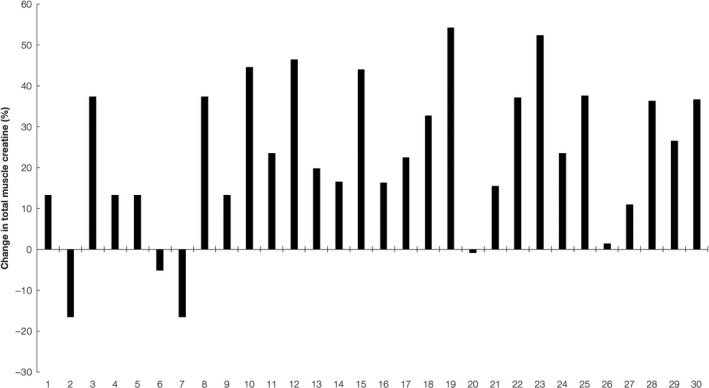
Individual changes in total muscle creatine (%) from baseline to 28‐day follow‐up for GAA trials

An average change in total creatine content after GAA supplementation was 22.9% (95% confidence interval 15.8–29.9), from 26.6 ± 4.6 mM at baseline to 32.2 ± 4.6 mM at follow‐up. Four subjects (13.3%) were categorized as nonresponders, two as quasi‐responders (6.6%), and 24 individuals (80.0%) were classified as responders (*p* < .001). For individual responses, a middle‐aged woman (39 years) experienced the highest increase in muscle creatine levels (54.2%) at postadministration, with 16 participants (53.3%) encountered an increase in total muscle creatine ≥20% (Figure 1). Contrarily, three participants (all men aged 22–25 years) experienced a reduction in muscle creatine (from 0.9% to 16.7%) at 28‐day follow‐up. Furthermore, a significant difference was found between groups in total creatine content at baseline (*p* = .003), with nonresponders have notably higher muscle creatine levels before GAA loading as compared to responders (33.0 ± 1.1 mM vs. 25.4 ± 4.1 mM; *p* = .001).

## 4 DISCUSSION

The current analysis of previously collected data describes a fairly high prevalence of individuals sensitive to dietary GAA (four out of five subjects), thus advancing this innovative agent as a rather effective tool to improve muscle creatine levels for at least 10% or more during 28‐day loading. Viewed in comparison with creatine trials, GAA loading appears to demonstrate a similar responder versus nonresponder performance. Nonresponders to creatine account for 20%–30% of the population (e.g., individuals showing less than a 10 mmol/kg dry mass [8%] increase in total muscle creatine after creatine loading) (Greenhaff, 1996) as compared to 20% of nonresponders plus quasi‐responders in GAA trial. However, a direct comparability remains open to debate due to significant differences in the type of intervention and specific dosages administered (e.g., loading phase with 20 g/day of creatine for 5–7 days followed by 3–5 g/day of creatine over the next 3 weeks vs. low‐dose creatine [2–3 g/day] for 1 month vs. 2–3 g/day of GAA for 4 weeks). GAA responders appear to have lower pretrial creatine levels, suggesting that those with unsaturated intramuscular creatine stores may better react to GAA treatment (e.g., vegetarians, elderly, women, supplement‐naïve population, and clinical patients). This is in accordance with previous creatine trials, reporting that individuals who have lower muscle creatine stores appear to experience a higher rise (20%–40%) in total muscle creatine, while those with relatively high muscle creatine levels may only increase half that amount or even less (Greenhaff, Bodin, Soderlund, & Hultman, 1994; Syrotuik & Bell, 2004). All nonresponders in our analysis with GAA loading have muscle creatine >31 mM, thus belonging to the upper quartile of our sample. Due to the small sample size, we were unable to adjust the responder analysis to participants age and gender, while both factors are known to affect GAA utilization (Joncquel‐Chevalier Curt et al., 2013). Hence, more adequately sampled studies are warranted before recommending GAA as a widely effective dietary additive in human nutrition. In particular, a detailed responder‐to‐nonresponder analysis for supplemental GAA to improve bioenergetics in other energy‐demanding tissues (*e.g.*, brain, liver, and myocardium) is highly justified due to the lack of useful and extensively applicable dietary interventions in the clinical environment.

## 5 STATEMENT OF ETHICS

The studies were approved by the local IRB at the University of Novi Sad, with the study protocols systematized in accordance with the World Medical Association Declaration of Helsinki.

## 6 INFORMED CONSENT

The participants signed an informed consent to voluntarily participate in all studies included in this report.

## CONFLICT OF INTEREST

The author reports no conflicts of interest associated with this manuscript.
